# A Heterozygous Missense Variant in the *COL5A2* in Holstein Cattle Resembling the Classical Ehlers–Danlos Syndrome

**DOI:** 10.3390/ani10112002

**Published:** 2020-10-30

**Authors:** Joana G. P. Jacinto, Irene M. Häfliger, Inês M. B. Veiga, Anna Letko, Cinzia Benazzi, Marilena Bolcato, Cord Drögemüller

**Affiliations:** 1 Department of Veterinary Medical Sciences, University of Bologna, 40064 Ozzano Emilia (Bologna), Italy; joana.goncalves2@studio.unibo.it (J.G.P.J.); cinzia.benazzi@unibo.it (C.B.); marilena.bolcato2@unibo.it (M.B.); 2 Institute of Genetics, Vetsuisse Faculty, University of Bern, 3012 Bern, Switzerland; irene.haefliger@vetsuisse.unibe.ch (I.M.H.); anna.letko@vetsuisse.unibe.ch (A.L.); 3 Institute of Animal Pathology, Vetsuisse Faculty, University of Bern, 3012 Bern, Switzerland; ines.veiga@vetsuisse.unibe.ch

**Keywords:** *Bos taurus*, collagen dysplasia, collagen V, connective tissue, precision medicine, skin fragility, whole-genome sequencing

## Abstract

**Simple Summary:**

Genodermatoses represent inherited disorders of the skin that mostly follow a monogenic mode of inheritance. Heritable connective tissue disorders such as classical Ehlers–Danlos syndrome (cEDS) belong to this group of human rare diseases that sporadically occur in other species. Herein, affected cattle are reported showing skin lesions including cutis laxa clinically and pathologically resembling cEDS in humans. Microscopic findings in the deeper dermis were consistent with collagen dysplasia. Whole-genome sequencing (WGS) identified a most likely disease-causing mutation in the *COL5A2* gene. The *COL5A2* gene is known to be associated with dominant inherited cEDS forms in mice and humans, but so far, it was not shown to cause a similar phenotype in domestic animals. The disease phenotype examined herein showed co-segregation with the identified missense variant within the maternal line across two generations and is most likely due to a spontaneous mutation event. Rare non-lethal disorders such as cEDS in livestock are mostly not diagnosed, but might affect animal welfare and thus lower the value of affected animals. WGS-based precision diagnostics allows understanding rare disorders and supports the value of surveillance of cattle breeding populations for harmful genetic disorders.

**Abstract:**

Classical Ehlers–Danlos syndrome (cEDS) is a heritable connective tissue disorder characterized by variable degrees of skin hyperextensibility and fragility, atrophic scarring, and generalized joint hypermobility. The purpose of this study was to characterize the clinicopathological phenotype of a cEDS-affected Holstein calf and to identify the causative genetic variant associated with the disorder by whole-genome sequencing (WGS). A 3-day-old female Holstein calf was referred because of easily induced skin detachment and hyperextensibility in the neck. A complete clinical investigation was performed in the calf, dam, and maternal-grandmother. The calf and dam showed hyperextensibility of the neck skin and atrophic scarring; additionally, the calf presented skin fragility. Moreover, the histopathology of biopsies from the calf and its dam showed that the collagen bundles in affected skin areas were wavy, short, thin, and surrounded by edema and moderate to severe acute hemorrhages. Genetic analysis revealed a private heterozygous missense variant in *COL5A2* (c.2366G>T; p.Gly789Val) that was present only in the calf and dam. This confirmed the diagnosis of cEDS and represents the first report of a causal variant for cEDS in cattle and the first *COL5A2*-related large animal model.

## 1. Introduction

Sporadically occurring, genodermatoses represent inherited disorders of the skin that mostly follow a monogenic mode of inheritance in livestock animals such as cattle [1]. Heritable connective tissue disorders, e.g., Ehlers–Danlos syndrome (EDS), belong to this group of human rare diseases. EDS encompasses a clinically- and heritably-heterogeneous group of connective tissue disorders (Online Mendelian Inheritance in Man (OMIM PS130000)) (https://www.omim.org/phenotypicSeries/PS130000) characterized by a variable degree of skin hyperextensibility, joint hypermobility, and tissue fragility. Currently, human EDS classification distinguishes 13 subtypes and 19 different associated genes mainly involved in collagen and extracellular matrix synthesis and maintenance reflecting the clinical and genetic heterogeneity [2]. Human EDS forms are grouped based on the underlying pathogenetic mechanisms related to primary structure and processing of collagen (*COL1A1*, *COL1A2*, *COL3A1*, *COL5A1, COL5A2*, *ADAMTS2*), collagen folding and cross-linking (*PLOD1*, *FKBP14*), structure and function of the myomatrix (*TNXB*, *COL12A1*), glycosaminoglycan biosynthesis (*B4GALT7*, *B3GALT6*, *CHST14*, *DSE*), complement pathway (*C1S*, *C1R*), and intracellular processes (*SLC39A13*, *ZNF469*, *PRDM5*) [3]. Classical EDS (cEDS) in humans is a rare autosomal dominant disorder predominantly associated with a deficiency of type V collagen (COLLV) encoded by the *COL5A1* and *COL5A2* genes, which is a quantitatively minor fibrillar collagen that presents a nearly ubiquitous distribution in a variety of connective tissues [4].

Various forms of EDS have been identified in many animal species (OMIA000327), including horses [5,6], dogs [7,8,9], cats [10], mink [11], rabbits [12], sheep [13,14,15], and cattle (OMIA000328-9913 (https://www.omia.org/OMIA000328/9913/); OMIA001716-9913 (https://www.omia.org/OMIA001716/9913/)) [16,17,18]. Pathogenic variants causing forms of EDS in animals have been identified in known candidate genes for EDS (*COL5A1*, *ADAMTS2*, *PLOD1*) [5,7,8,10,13,15,18], or novel genes (*EPYC, TNBX, PPIB)* discovered in EDS-affected domestic animals [6,9,17]. This highlights the potential of studying inherited conditions in such species to assign a role or function to previously uncharacterized genes or to add additional functions to known genes in regard to skin development [1].

In this study, we aimed to characterize the clinical and pathological phenotypes of a cEDS-affected Holstein calf and its dam, and to identify the causative genetic variant associated with the disorder using whole-genome sequencing (WGS).

## 2. Materials and Methods

### 2.1. Ethics Statement

This study did not require official or institutional ethical approval as it was not experimental, but rather part of clinical and pathological veterinary diagnostics. All animals in this study were examined with the consent of their owners and handled according to good ethical standards.

### 2.2. Clinicopathological Investigation

A 3-day-old female Holstein calf was referred by the farm veterinarian because of easily induced skin detachment in the neck and skin hyperextensibility shortly after birth. Upon specific request, the owner informed that its 3-year-old dam and its 6-year-old maternal-grandmother had also been presenting skin alterations for a long time, but was not able to specify since when. All three animals, the calf, its dam, and its maternal-grandmother, were clinically examined and a complete blood count (CBC) and blood chemistry profile were obtained. Blood samples from the calf and its dam were sent for routine viral and parasitological analysis (bovine viral diarrhea, bovine Schmallenberg virus, bluetongue virus, *Neospora* spp., *Toxoplasma* spp.) using antigen-enzyme-linked immunosorbent assay (ELISA) and antibody-polymerase chain reaction (PCR). Three weeks later (calf’s age = 29 days), skin biopsies using an 8 mm punch were obtained from the ulcerated cervical skin, from the skin surrounding the cervical ulceration, and from normal skin from the neck from the calf, as well as from the altered cervical skin from its dam. The collected samples were fixed in 10% neutral buffered formalin, trimmed, processed, embedded in paraffin wax, sectioned at 4 µm, and stained with haematoxylin and eosin (H&E) for further histological evaluation. Further clinical control was carried out after six months (calf’s age = 7 months). All animals were housed in a freestall system.

### 2.3. DNA Samples

Genomic DNA was isolated from ethylenediaminetetraacetic acid (EDTA) blood samples from the calf and its dam; from EDTA blood sample, normal skin, and lesioned skin of the maternal-grandmother; and from the semen of the sire using Promega Maxwell RSC DNA system (Promega, Dübendorf, Switzerland).

### 2.4. Whole-Genome Sequencing

WGS using the Illumina NovaSeq6000 (Illumina Inc., San Diego, CA, USA) was performed on the genomic DNA of the calf. The sequenced reads were mapped to the ARS-UCD1.2 reference genome, resulting in an average read depth of approximately 17× [19], and single-nucleotide variants (SNVs) and small indel variants were called. The applied software and steps to process fastq files into binary alignment map (BAM) and genomic variant call format files were in accordance with the 1000 Bull Genomes Project processing guidelines of run 7 (www.1000bullgenomes.com) [20], except for the trimming, which was performed using fastp [21]. Further preparation of the genomic data was done according to Häfliger et al., 2020 [22]. In order to find private variants, we compared the genotypes of the affected calf with 496 cattle genomes of various breeds that had been sequenced in the course of other ongoing studies and that are publicly available (Table S1) in the European Nucleotide Archive (SAMEA7015115 is the sample accession number of the affected calf; http://www.ebi.ac.uk/en). Integrative Genomics Viewer (IGV) [23] software was used for visual inspection of genome regions containing possible candidate genes.

### 2.5. Targeted Genotyping

Polymerase chain reaction (PCR) and Sanger sequencing were used to validate and genotype the variant identified from WGS. PCR products from genomic DNA were amplified using AmpliTaq Gold 360 Master Mix (Thermo Fisher Scientific, Waltham, MA, USA) and the PCR amplicons were directly sequenced on an ABI3730 capillary sequencer (Thermo Fisher Scientific, Darmstadt, Germany). The *COL5A2* missense variant (XM_024979774.1:g.7331916G>T) was genotyped using the following primers: 5’- ACCAGGGCTTCAAGGTATGC-3’ (forward primer) and 5’-CACCATGGGAACATGAGGCT-3’ (reverse primer). The sequence data were analyzed using Sequencher 5.1 software (GeneCodes, Ann Arbor, MI, USA).

### 2.6. Protein Predictions

PROVEAN [24], MutPred2 [25], and PredictSNP1 [26] were used to predict the biological consequences of the discovered variant on protein. For multispecies sequence alignments, the following National Center for Biotechnology Information (NCBI) proteins accessions were used: XP_024835542.1 (*Bos taurus*), NP_000384.2 (*Homo sapiens*), XP_001164152.1 (*Pan troglodytes*), XP_002799008.1 *(Macaca mulatta*), XP_005640450.1 (*Canis lupus*), NP_031763.2 (*Mus musculus*), NP_445940.1 (*Rattus norvegicus*), XP_004942453.1 (*Gallus gallus*), NP_001139254.1 (*Danio rerio*), and XP_002931546.2 (*Xenopus tropicalis*).

### 2.7. Sequence Accessions

All references to the bovine *COL5A2* gene correspond to the NCBI accessions NC_037329.1 (chromosome 2, ARS-UCD1.2), XM_024979774 (*COL5A2* mRNA), and XP_024835542.1 (COL5A2 protein). For the protein structure of COL5A2, the Uniprot database (https://www.uniprot.org/) accession number A0A3Q1MDT9 was used.

## 3. Results

### 3.1. Clinical Phenotype

On clinical investigation, the calf, its dam, and its maternal-grandmother were found to be clinically healthy with the exception of the skin alterations. Particular clinical examination of the cardiovascular, respiratory, urinary, musculoskeletal, and nervous systems showed no abnormalities. Moreover, no joint hypermobility was observed. The blood investigation of the calf showed a moderate monocytosis (1760/mm^3^), a mild neutrophilia (4650/mm^3^), a mild hypocholesterolemia (47 mg/dL), and a mild hypoproteinemia (6.10 g/dL) with hypoalbuminemia (2.82 g/dL). No abnormalities were detected in the CBC and chemistry profiles of the dam and maternal-grandmother. Blood viral analysis revealed positivity for bovine Schmallenberg virus using antigen-ELISA in the calf, and positivity for bovine viral diarrhea using antigen-ELISA in the calf and its dam. The animals tested negative for all the remaining viral and parasitological analyses.

**Figure 1:**
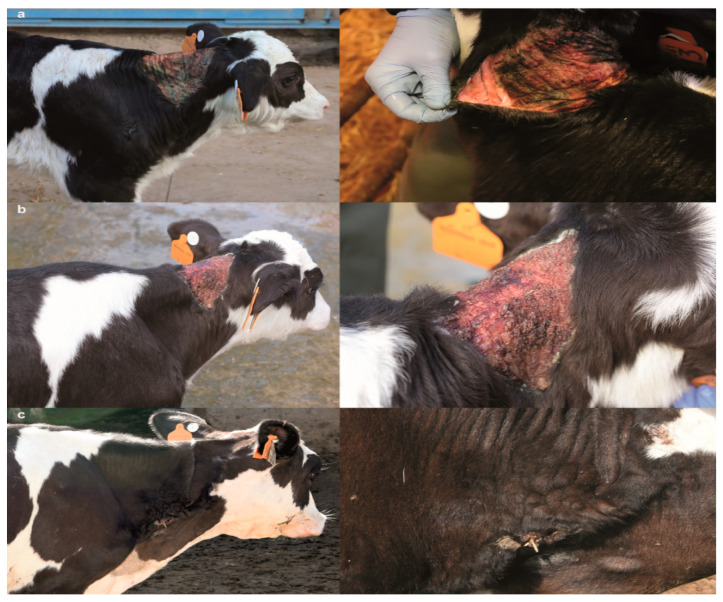
Neck skin lesions of the classical Ehlers–Danlos syndrome (cEDS)-affected Holstein calf. (**a**) Calf age = 3 days: severe, extensive ulceration secondary to minor trauma; note the spontaneous detachment of the skin from the subcutaneous tissue. (**b**) Calf age = 29 days: atrophic scarring; note the scabby and crustose wound with the edges firmly embedded in the subcutaneous tissue. (**c**) Calf age = 7 months: papyraceous scars and cutis laxa.

The integumentary system examination at 3 days of age of the calf revealed a symmetrical, bilateral ulceration secondary to minor trauma, delimitated cranially by the occipital region, caudally by the cranial margin of the scapula, and ventrally by the sternocephalic muscles (Figure 1a). The ulcerated surface was dry, non-painful, non-bleeding, and pinkish with the presence of purulent material at the edges. During palpation, at the wound edges, a spontaneous detachment of the skin from the subcutaneous tissue was noticed. Furthermore, the animal presented hyperextensibility of the skin mostly in the neck. At 29 days of age, the previously observed neck wound was dry, scabby, and crustose with the edges of the lesion firmly embedded in the subcutaneous tissue; atrophic scarring was also present (Figure 1b). At seven months, the calf’s lesions were similar to its dam, and characterized by multiple wrinkles, folds, papyraceous scars, cutis laxa, and hyperextensibility of the neck skin (Figure 1c).

**Figure 2:**
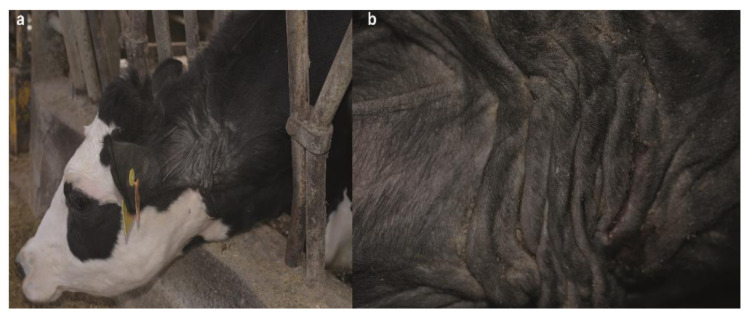
Neck skin lesions of the cEDS-affected Holstein dam. (**a**) Note the multiple wrinkles, folds, papyraceous scars and cutis laxa. (**b**) Details of the neck skin lesion.

Examination of the dam revealed multiple wrinkles, folds, papyraceous scars, cutis laxa, and hyperextensibility of the neck skin (Figure 2a,b). Moreover, its maternal-grandmother showed milder skin lesions in the ventral part of the neck at the level of the larynx, characterized by the presence of bald areas and scabs (Figure S1).

Based on these clinical observations, the calf and its dam were consequently suspected to suffer from cEDS, while the skin lesions observed in the maternal-grandmother were suspected to be acquired as the skin has been traumatised by recurring long-term mechanical stress of the feed fence.

### 3.2. Histopathological Phenotype

**Figure 3:**
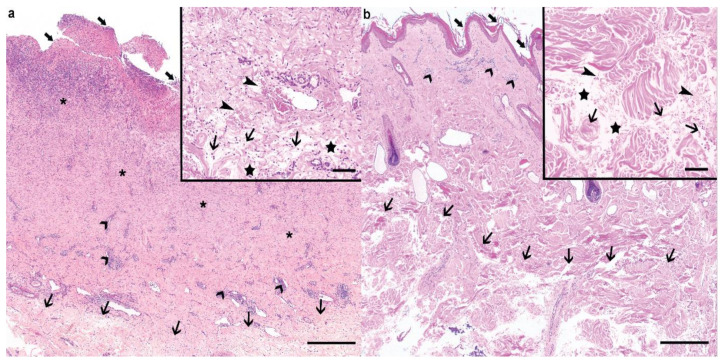
Histological findings of the cEDS-affected Holstein calf and its dam. (**a**) The ulcerated (large arrows) cervical lesion from the calf displayed a prominent granulation tissue proliferation (asterisks) associated with neovascularization and severe neutrophilic infiltration. Moderate, lymphoplasmacytic perivascular infiltrates were visible in the superficial dermis (large arrowheads). Within the deeper dermis, the collagen bundles were loose and irregular (thin arrows). Haematoxylin and eosin (H&E), bar 500 µm. Inset: higher magnification of the affected connective tissue within the deeper dermis. The collagen bundles were wavy, short, and thin (thin arrows), and surrounded by edema (stars) and acute hemorrhage (thin arrowheads) in the absence of vascular changes. Haematoxylin and eosin (H&E), bar 100 µm. (**b**) In the dam, the epidermis was irregular, mildy hyperplastic, and layered by large amount of lamellar to compact, orthokeratotic keratin (large arrows). Mild to moderate, perivascular lymphocytic and plasmacellular infiltrates were visible in the superficial dermis (large arrowheads), while similar changes to the ones described in the calf could be observed in the deeper dermis (thin arrows). Haematoxylin and eosin (H&E), bar 500 µm. Inset: Higher magnification of the affected connective tissue within the deeper dermis. Similar changes to the ones observed in the calf could be observed, namely, wavy, short, and thin collagen bundles (thin arrows), interstitial edema (stars), and acute hemorrhage (thin arrowheads). Haematoxylin and eosin (H&E), bar 100 µm.

Histologically, the epidermis at the level of the cervical lesion of the calf displayed a severe, diffuse ulceration, with underlying proliferation of mature granulation tissue associated with neovascularisation, abundant neutrophilic superficial infiltrates, and adnexal structure loss (Figure 3a). The epidermis at the border of the ulcerated area displayed abundant serocellular crusts associated with serum lake formation, spongiosis, and ballooning degeneration of the stratum granulosum. Multifocal perivascular, moderate lymphoplasmacytic infiltrates could be observed in the dermis underlying the ulcerated area. The deeper dermis displayed interlacing, wavy, short, thin collagen bundles, which were surrounded by a moderate interstitial edema, as well as moderate to severe acute hemorrhages (Figure 3a).

In the punch biopsy taken from the normal skin from the neck, the epidermis displayed a normal thickness and was covered by a large amount of fairly compact, orthokeratotic keratin. Mild to severe, interstitial eosinophilic and neutrophilic infiltrates of unknown origin were present mostly within the deeper dermis and did not allow the identification of the dermal changes observed at the site of the ulceration.

The cutaneous punch biopsy taken from the neck of the dam displayed a mildly hyperplastic and irregular epidermis, and was covered by a large amount of lamellar to compact, orthokeratotic keratin (Figure 3b). The superficial dermis displayed mild to moderate perivascular infiltrates composed of lymphocytes, plasma cells, and occasionally eosinophils. The deeper dermis displayed similar changes to the ones observed in the biopsies from the calf (Figure 3b).

The histological findings in the deeper dermis from both the calf and its dam were compatible with collagen dysplasia within the deeper dermis, and thus with the clinical suspicion of cEDS.

### 3.3. Genetic Analysis

**Figure 4:**
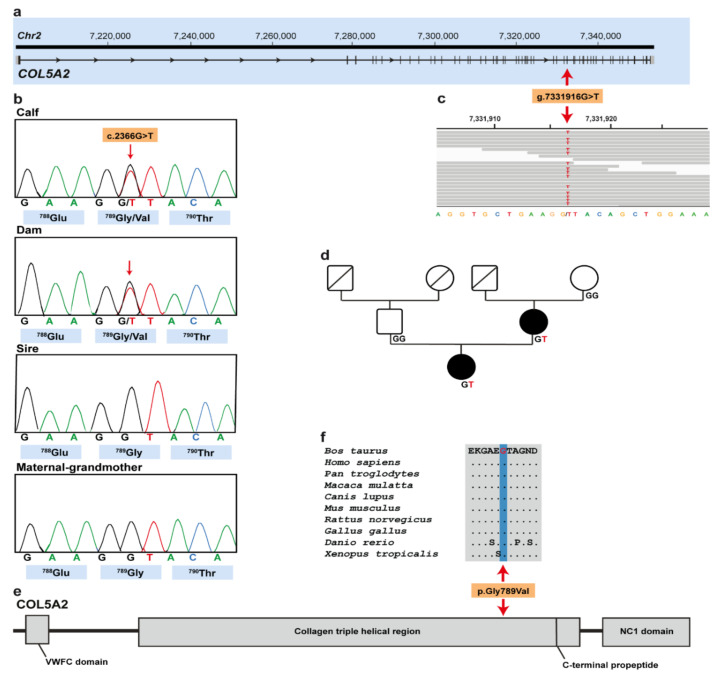
cEDS-associated *COL5A2* missense variant in Holstein cattle. (**a**) *COL5A2* gene structure showing the variant location on chromosome 2, exon 35 (red arrow). (**b**) Electropherograms of the calf, dam, sire, and maternal-grandmother. (**c**) Integrative Genomics Viewer (IGV) screenshot presenting the g.7331916G>T variant in the affected calf. (**d**) Pedigree of the cEDS-affected Holstein family. Males are represented by squares and females by circles. Affected animals are represented with full black symbols, while non-affected animals are represented by full white symbols. Unknown genotypes are represented by symbols with a diagonal line. *COL5A2* genotypes are shown for all available animals. (**e**) Schematic representation of COL5A2 protein and its three functional domains. (**f**) Multiple sequence alignment of the collagen alpha-2(V) chain of the COL5A2 protein encompassing the region of the p.Gly789Val variant demonstrates complete evolutionary conservation across species.

Filtering of WGS for private variants present in the affected calf and absent in the 496 available control genomes identified a single protein-changing variant in COL5A2 with a predicted moderate impact on the encoded protein. The heterozygous variant in COL5A2 exon 35 on chromosome 2 (chr2:g.7331916G>T) was confirmed using IGV software (Figure 3a,c). The detected COL5A2 variant (XM_024979774.1: c.2366G>T) alters the encoded amino acid of COL5A2 residue 789 of the collagen triple-helical region (XP_024835542.1: p.Gly789Val) included in the collagen alpha-2(V) chain (Figure 4e). Furthermore, the glycine to valine substitution affects an evolutionary conserved residue (Figure 4f) and was predicted to be deleterious (PROVEAN score: −8.294; MutPred2 score: 0.917; PredictSNP1 score: 0.869). To confirm and evaluate the presence of the COL5A2 variant, the affected genomic region was amplified by PCR and Sanger sequenced in the calf, its dam, its maternal-grandmother, and its sire (Figure 4b). Analyzing the sequencing data, we observed that the calf and its dam were heterozygous for the detected COL5A2 variant, whereas the sire and the maternal-grandmother were homozygous for the wild type allele (Figure 4b,d). Unfortunatly, no samples from other closely related animals such as the maternal-grandfather were available.

## 4. Discussion

The identified missense *COL5A2* variant affects a functionally important site of an obvious candidate gene and thus represents the most likely pathogenic variant associated with the observed cEDS phenotype of two examined Holstein cattle family members. In veterinary medicine, so far, two distinct pathogenic variants in *COL5A1* associated with cEDS have been reported in dogs [8] and cats [10]. To the best of our knowledge, no pathogenic variant in the *COL5A2* associated with cEDS has been reported in domestic animal species. Therefore, this study in cattle provides the first example of a *COL5A2*-related congenital skin disorder in domestic animals.

In human medicine, mutations in *COL5A2* are associated with autosomal dominantly inherited cEDS type 2 (OMIM 130010) (https://omim.org/entry/130010). The most recent update of the Leiden Open Variation Database (LOVD) lists 312 different pathogenic variants that affect COLLV [27]. In particular, 220 distinct *COL5A1* and 92 *COL5A2* pathogenic variants are described [28]. Diagnosis of cEDS in humans relies on fulfilling minimal criteria that encompass skin hyperextensibility and atrophic scaring plus either another major criterion, generalized joint hypermobility, and/or at least three minor criteria (Table 1), as well as mandatory molecular test confirmation [2].

As described before, the calf and its dam carry a deleterious heterozygous missense variant in the *COL5A2* gene. More than 90% of cEDS human patients harbor a heterozygous mutation in one of the genes encoding COLLV [4,29,30], such as, for example, the missense variant p.Gly934Arg in *COL5A2*, where there is a substituition of a glycine residue within the triple helical domain (Gly-X-Y) [31].

The clinical and pathological phenotype in both cases of this study resembled a form of cEDS. The affected Holstein calf and its dam met one major criterion (skin hyperextensibility and atrophic scarring) of the human classification system (Table 1). Furthermore, one minor criterion was met in the calf (skin fragility/traumatic splitting). Even though the two presented cases do not completely fulfill the clinical criteria for human cEDS classification, the diagnosis of cEDS has been assumed. It is worth noting that, in veterinary medicine, just a few cases have been reported in the literature, rendering it difficult to develop such a classification system adapted for domestic animal species. In addition, the cases presented herein show lesions restricted to the neck region. Therefore, this may be a specific characteristic of bovine cEDS. Furthermore, it is assumed that the skin lesions observed in the maternal-grandmother were linked to trauma. In fact, the prevalalence of neck skin lesions related to the infrastructure of freestall farms is around 9% [32]. Environmental factors cause phenocopies, which are incidents in which non-genetic conditions simulate a genetic disorder. On the farm where the three animals were housed, there were more cows showing neck skin lesions similar to the maternal-grandmother’s that may represent phenocopies due to recurring long-term mechanical stress of the feed fence. However, the calf’s owner did not recall having any similarly affected animals in the past as the cEDS-affected calf and its dam.

We speculate that the mutation either occurred post-zygotically during the fetal development of the affected dam or represents a germline mutation that occurred in the maternal-grandfather. Nonetheless, this *de novo* mutation was then transmitted to the cEDS-affected calf. The amplification of the mutated allele in the maternal-grandmother using DNA extracted from EDTA blood, skin from the neck lesions area, and normal skin resulted in homozygous wild-type status. Therefore, the maternal-grandmother has been excluded as a mosaic ancestor. However, to prove that the identified mutation in *COL5A2* indeed occured *de novo*, genotyping of the maternal-grandsire would be needed.

Moreover, the identified deleterious variant and the conservation of the affected glycine amino acid residue of COL5A2 at position 789 in the highly conserved triple-helical domain also suggest that this variant is most likely pathogenic. The predicted amino acid exchange occurs in the protein triple-helical domain, and by analogy with helix glycine substitutions; for example, in collagen alpha-1(I) in osteogenesis imperfecta (OMIM 120150) (https://www.omim.org/entry/120150), in collagen alpha-2(II) in chondrodysplasias (OMIM 120160) (https://www.omim.org/entry/120160), and in collagen alpha-3(III) and -4(IV) in Alport syndrome II (OMIM 203780) (https://www.omim.org/entry/203780), it would be expected to disrupt the propagation of collagen triple helix, resulting in abnormal molecules linked to the disorder [33]. Collagen triple helix folding and stability is critically dependent on having a glycine as every third amino acid in the triplet repeat sequence (Gly-x-y). Therefore, replacing glycine with a bulky amino acid (in this case, a valine) has the potential to disrupt helix folding and lead to increased posttranslational lysine hydroxylation and glycosylation, compromising the triple helix structural integrity and retention of the mutant trimers in the endoplasmic reticulum, which can have an impact in the cellular function [34]. In addition, cellular quality control mechanisms that bring about endoplasmic reticulum-retention and degradation of misfolded collagens are leaky and collagen hetero- or homotrimers containing one or several mutant pro-alpha-chains are often secreted, having an important predicted impact on collagen fibril formation and stability, and altered interactions with other extracellular matrix components [34]. The major variant of COLLV is a heterotrimer composed of two pro-alpha-1 chains and a single pro-alpha-2 chain, which are encoded by the *COL5A1* and *COL5A2* genes, respectively [35]. COLLV plays a central role in the assembly of tissue-specific matrices. Several COLLV isoforms have been reported, however, the most widely accepted form is the [α1(V)]2α2(V) heterotrimer that co-assembles with type I collagen into heterotypic type I/V collagen fibrils in the extracellular matrix. COLLV is thought to regulate the diameter of these fibrils by retention of its large N-propeptide domain, which projects above the surface of the collagen fibril [35].

## 5. Conclusions

Rare non-lethal disorders such as cEDS in livestock are usually not reported or diagnosed when the animals show mild to moderate phenotype, but they affect animal welfare through secondary wounds and thus lower the value of the affected animals. Additionally, molecular diagnosis is often not performed because of a lack of resources and diagnostic tools, and/or low value of the animals.

Investigation of these cases allowed a complete clinical, pathological, and molecular genetic study, enabling for the first time the diagnosis of a dominantly inherited cEDS form in a family of Holstein cattle associated with a *COL5A2* variant. Furthermore, this example highlights the utility of WGS-based precision diagnostics for understanding rare disorders in animals with an available reference genome sequence and the value of surveillance of cattle breeding populations for harmful genetic disorders.

## Figures and Tables

**Table 1:** Classification of human classical Ehlers–Danlos syndrome (cEDS).

**Inheritance**	Autosomal dominant
**Molecular Basis**	*COL5A1*; *COL5A2; COL1A1; COL3A1*
**Major Criteria**	1. Skin hyperextensibility and atrophic scarring
	2. Generalized joint hypermobility
**Minor Criteria**	1. Easy bruising
	2. Soft, doughy skin
	3. Skin fragility (or traumatic splitting)
	4. Molluscoid pseudotumors
	5. Subcutaneous spheroids
	6. Hernia
	7. Epicanthal folds
	8. Complications of joint hypermobility
	9. Family history of first-degree relative who meets clinical criteria

Adapted from Malfait et al. 2017 [2].

## Supplementary Materials

The following are available online at https://www.mdpi.com/2076-2615/10/11/2002/s1, Table S1: Project and Sample ID for public access of the whole genome sequenced genomes used in this study. EBI Accession numbers of all publicly available genome sequences. We compared the genotypes of the calf with 496 cattle genomes of various breeds that had been sequenced in the course of other ongoing studies and that were publicly available. Figure S1: Neck skin lesions of the maternal-grandmother. Note the presence of bald areas and scabs.
