# Supplementary figures and images for: A Heterozygous Missense Variant in the COL5A2 in Holstein Cattle Resembling the Classical Ehlers–Danlos Syndrome
Source: Animals (Basel). 2020 Oct 30;10(11):2002. doi: 10.3390/ani10112002 (PMC7692662; doi:10.3390/ani10112002)

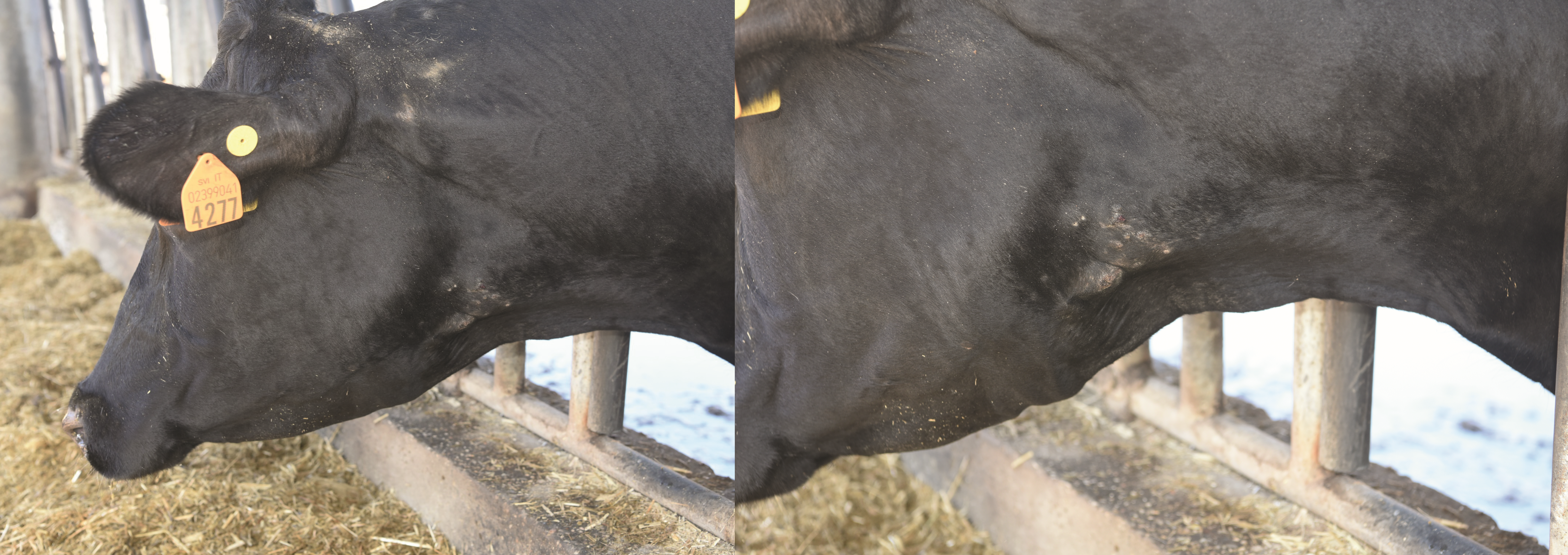

Supplement: Supplementary file 1 [file animals-10-02002-s001.zip › Figure S1.tif]
